# Beyond *H. pylori*: Re-Examining the Oral Microbiome’s Role in Gastric Health and Disease, a Narrative Review

**DOI:** 10.3390/medicina61122222

**Published:** 2025-12-16

**Authors:** Diana Tatarciuc, Dragos Catalin Ghica, Mioara Darnea, Irina Mihaela Esanu, Roxana-Ionela Vasluianu, Ovidiu Stamatin, Lucian Indrei, Magda Antohe, Iulian-Costin Lupu, Livia Bobu, Ana Maria Dima

**Affiliations:** 1 Department of Medical Semiology, Faculty of Medicine, Grigore T. Popa University of Medicine and Pharmacy, 700115 Iasi, Romania; diana.tatarciuc@umfiasi.ro (D.T.); irina.esanu@umfiasi.ro (I.M.E.); 2 Department of Preventive Medicine and Interdisciplinarity, Faculty of Medicine, Grigore T. Popa University of Medicine and Pharmacy, 700115 Iasi, Romania; dragos.ghica@yahoo.ro (D.C.G.); lucian.indrei@umfiasi.ro (L.I.); 3 Department of Prosthodontics, Faculty of Dental Medicine, “Dimitrie Cantemir” University Targu Mures, 504545 Târgu Mureș, Romania; miodar2@yahoo.com; 4 Department of Prosthodontics, Faculty of Dental Medicine, “Grigore T. Popa” University of Medicine and Pharmacy, 700115 Iasi, Romania; ovidiu.stamatin@umfiasi.ro (O.S.); magda.antohe@umfiasi.ro (M.A.); iulian.lupu@umfiasi.ro (I.-C.L.); 5 Department of Surgicals, Faculty of Dental Medicine, “Grigore T. Popa” University of Medicine and Pharmacy, 700115 Iasi, Romania; livia.bobu@umfiasi.ro; 6 Independent Researcher, 700115 Iasi, Romania; amadi2024@proton.me

**Keywords:** oral-systemic link, periodontitis, gastritis, *Helicobacter pylori*, oral microbiome, gastric microbiome, systemic inflammation, dysbiosis, interdisciplinary care, nitrate metabolism

## Abstract

*Background*: The separation between oral and systemic health is increasingly challenged. Globally prevalent inflammatory diseases such as gastritis, often caused by *Helicobacter pylori (H. pylori)*, and oral pathologies like periodontitis may be interconnected through microbial and inflammatory pathways. *Objective*: This review synthesizes evidence on the dental-gastric link, examining mechanistic pathways and clinical implications. *Methods*: A structured literature search identified key studies from 2000 to 2025, prioritizing systematic reviews and high-quality human research. *Findings*: Three key mechanistic pathways link oral dysbiosis with gastric pathology: (1) the direct translocation of oral pathogens to the stomach, including *H. pylori* and the broader dysbiotic oral microbiome; (2) the systemic inflammatory spillover from the periodontium, which primes the host immune system and exacerbates gastric inflammation; and (3) ancillary mechanisms such as the disruption of beneficial nitrate-nitrite-nitric oxide metabolism. Epidemiological studies show strong associations, and initial interventional trials indicate periodontal therapy may improve *H. pylori* eradication rates and reduce recurrence. However, the evidence is tempered by methodological limitations, including profound confounding by shared risk factors (e.g., smoking, socioeconomic status), the challenge of reverse causality, and inconsistent results from interventional studies. *Conclusion*: While confounding factors require consideration, oral health is a promising modifiable risk factor for gastritis. Interdisciplinary collaboration between dentistry and gastroenterology is essential to advance research and integrate oral care into gastrointestinal disease management.

## 1. Introduction: The Oral-Gastric Axis in Health and Disease

For centuries, the medical and dental professions have operated in relative silos, with the oral cavity often considered a separate entity from the rest of the human body. This artificial dichotomy is rapidly dissolving under the weight of compelling evidence establishing oral health as a critical determinant of systemic well-being [1,2,3,4]. The concept of the “oral-systemic link” has evolved from a hypothesis into a cornerstone of modern pathophysiology, implicating oral infections in a spectrum of diseases including cardiovascular ailments, diabetes mellitus, rheumatoid arthritis, and adverse pregnancy outcomes [5,6,7,8]. Within this expansive landscape, the intricate connection between the oral cavity and the gastrointestinal tract represents one of the most direct and physiologically plausible pathways, yet it remains underexplored in its complexity [9,10]. This review focuses on a specific and clinically significant component of this pathway: the potential bidirectional relationship between dental pathologies, primarily periodontitis and caries, and gastritis. While some frameworks approach this topic by detailing each site’s microbiome separately, this review is structured around the functional pathways of their interaction to provide a mechanistic perspective on the oral-gastric axis.

Gastritis, the inflammation of the gastric mucosa, represents a global health burden of staggering proportions. It is primarily driven by chronic infection with *Helicobacter pylori* (*H. pylori*), a Gram-negative bacterium that colonizes the stomachs of over half the world’s population [11]. While many infections are asymptomatic, chronic *H. pylori*-induced gastritis is a well-established precursor to peptic ulcer disease, gastric mucosa-associated lymphoid tissue (MALT) lymphoma, and gastric adenocarcinoma, the latter remaining the fifth most common cancer and the fourth leading cause of cancer-related deaths worldwide [12,13,14]. The pathogenesis of *H. pylori* is complex, involving bacterial virulence factors (e.g., CagA, VacA), host immune responses, and environmental co-factors [15]. Despite the efficacy of combination antibiotic therapies, eradication failure and recurrence remain significant clinical challenges, suggesting the existence of reservoirs outside the gastric niche that can facilitate reinfection [16,17,18].

The Oral Ecosystem: The oral ecosystem in health is characterized by a diverse and balanced community of commensal bacteria, maintained in equilibrium by host immunity. Nevertheless, in states of dysbiosis such as periodontitis and caries, this symbiosis collapses. Periodontitis, in particular, represents a canonical shift to a pathogenic state, driven by ecological pressures that select for a less diverse, more virulent microbiota [19,20]. This dysbiotic community is dominated by proteolytic, pro-inflammatory pathobionts like *Porphyromonas gingivalis* and *Tannerella forsythia* (the “red complex”), which manipulate host immunity, leading to chronic inflammation and the destruction of periodontal tissues [21,22,23,24]. This creates a state of perpetual oral dysbiosis and a compromised ecological barrier [25,26].

The Gastric Ecosystem: In contrast, the gastric environment presents a formidable challenge for microbial colonization due to its acidic pH. Despite this, it hosts a unique microbiome and is the primary niche for *Helicobacter pylori* (*H. pylori*), the major driver of gastric pathology [11]. Chronic *H. pylori* infection induces gastritis through a complex interplay of bacterial virulence factors (e.g., CagA, VacA) and the host immune response, establishing a well-defined pathway to peptic ulcer disease, gastric MALT lymphoma, and gastric adenocarcinoma [12,13,14,15]. The persistent challenges of eradication failure and recurrence suggest the existence of extra-gastric reservoirs that can facilitate reinfection [16,17,18].

The theoretical and physiological basis for a link between these two prevalent conditions is robust. The gastrointestinal tract begins in the oral cavity, and the continuous swallowing of saliva (approximately 1.5 L per day) ensures a constant influx of oral microorganisms and inflammatory mediators into the stomach [27,28,29]. This establishes a direct “oral-gastric axis” where the oral environment can directly influence the gastric ecosystem. The historical and most straightforward hypothesis posits the oral cavity, particularly dental plaque, as a potential extra-gastric reservoir for *H. pylori* [30,31,32]. Numerous studies have detected *H. pylori* DNA and specific antigens in dental plaque and saliva using polymerase chain reaction (PCR), culture, and immunohistochemistry, with prevalence rates varying widely, often higher in individuals with poor oral hygiene and periodontal disease [33,34]. This reservoir theory is clinically significant, as it could explain instances of failed eradication therapy and recurrent gastric infection, hypothesizing that oral *H. pylori* can recolonize the stomach after antibiotic clearance [35,36].

However, to confine the investigation to *H. pylori* alone is to overlook a far more complex and potentially broader mechanistic landscape. The advent of high-throughput sequencing technologies has revolutionized our understanding of human-associated microbiomes, revealing that the oral and gastric microbiomes, while distinct, are intimately connected [37,38,39]. Periodontitis is not merely an infection by a few select pathogens but a state of profound microbial dysbiosis where the diversity of the oral microbiome collapses, and pathobionts thrive [40,41]. The swallowing of this dysbiotic microbiota represents a daily inoculation of the stomach with a complex consortium of pro-inflammatory pathogens and their virulence factors (e.g., lipopolysaccharides, gingipains). While the stomach’s acidic environment is a formidable barrier, many oral bacteria, including periodontopathogens, possess acid tolerance mechanisms and can survive transit, potentially acting as pathobionts that could disrupt gastric homeostasis and exacerbate local inflammation [42,43,44].

Beyond the direct translocation of bacteria, a second, parallel pathway involves systemic inflammation. Periodontitis is a significant source of low-grade, chronic systemic inflammation. The local inflammatory process in the periodontium leads to increased serum levels of pro-inflammatory cytokines (e.g., C-reactive protein, IL-1β, IL-6, TNF-α) and inflammatory mediators [45,46,47,48]. This systemic “inflammatory priming” can alter the host’s immune status, potentially lowering the threshold for inflammatory responses in other tissues, including the gastric mucosa. This could render the stomach more susceptible to damage from *H. pylori* or other pathogenic bacteria, amplifying the severity of gastritis [49,50].

Historically, the investigation of oral-gastric connections has been dominated by a pathogen-centric model, focusing almost exclusively on *Helicobacter pylori* and the hypothesis of the oral cavity as an extra-gastric reservoir. This paradigm, while foundational, offers a limited perspective by isolating a single pathogen from the complex microbial ecosystems of both sites. This review aims to contextualize and then move beyond this established model by synthesizing evidence for a broader holistic microbiome paradigm. This emerging perspective considers the collective impact of the entire oral microbial community—its state of dysbiosis, its diverse metabolic output, and its role as a continuous inoculum for the gut—as a critical factor influencing gastric health and disease. The following chapters will first examine the evidence for the classic *H. pylori* reservoir hypothesis (Section 3) before expanding the discussion to explore the modern, multi-faceted oral-gastric axis in the microbiome era (Section 4, Section 5 and Section 6).

**Figure 1:**
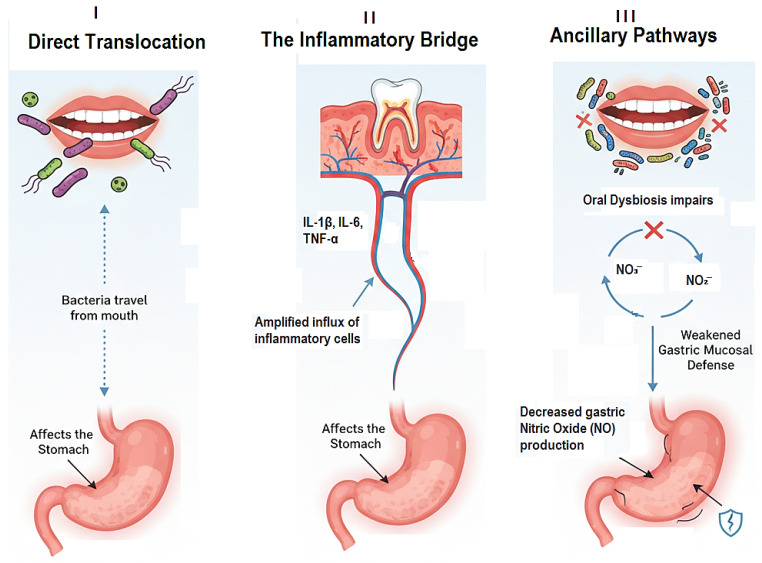
The Three Core Mechanistic Pathways Linking Oral Dysbiosis to Gastritis.

Therefore, the objective of this narrative review is to move beyond the established reservoir hypothesis and synthesize the current evidence investigating the multifactorial link between dental pathologies and gastritis. It evaluates the epidemiological evidence connecting periodontitis and caries to gastritis and its sequelae. It delves into the modern microbiome perspective, exploring the concept of oral-gut microbiome dysbiosis and its consequences for gastric health. Furthermore, it dissects the mechanisms spanning direct bacterial translocation and systemic inflammation. By integrating evidence from microbiology, immunology, gastroenterology, and dentistry, this review aims to provide a comprehensive up-to-date assessment, highlight critical knowledge gaps, and propose future research directions to validate this compelling oral-gastric axis. Establishing this link could have profound implications for the interdisciplinary management of patients, positioning oral health intervention as a novel adjunctive strategy in preventing and managing gastritis and its devastating consequences (Figure 1).

## 2. Materials and Methods

To ensure a comprehensive synthesis of the current evidence, an extensive literature search and analysis strategy was employed. This methodology was designed to capture the highest quality and most relevant evidence linking dental pathologies to gastritis.

### 2.1. Search Strategy

A comprehensive research of the literature was conducted using the electronic databases PubMed/MEDLINE, Web of Science, and Scopus for articles published from January 2000 to 2025. Key search concepts included (“periodontitis” OR “oral microbiome”) AND (“gastritis” OR “Helicobacter pylori” OR “gastric cancer”) to capture studies on association and mechanism. The search was iterative, and reference lists of retrieved articles were screened to ensure comprehensive coverage.

### 2.2. Study Selection and Eligibility Criteria

The focus was on identifying human studies published in English. The selection process prioritized evidence in the following hierarchy:

- Systematic Reviews and Meta-Analyses: Given their role in synthesizing high-level evidence.
- Randomized Controlled Trials (RCTs): Particularly for evaluating interventional evidence (e.g., impact of periodontal therapy on *H. pylori* eradication).
- Prospective Cohort and Case–Control Studies: For assessing temporal relationships and risk.
- Large Cross-Sectional Studies: For establishing associations, acknowledging the inherent limitations of this design.

Studies were excluded if they were editorials, case reports, conference abstracts only, or focused on animal or in vitro models without a clear link to human clinical outcomes.

## 3. The *H. pylori* Paradigm: The Oral Cavity as an Extra-Gastric Reservoir

**Figure 2:**
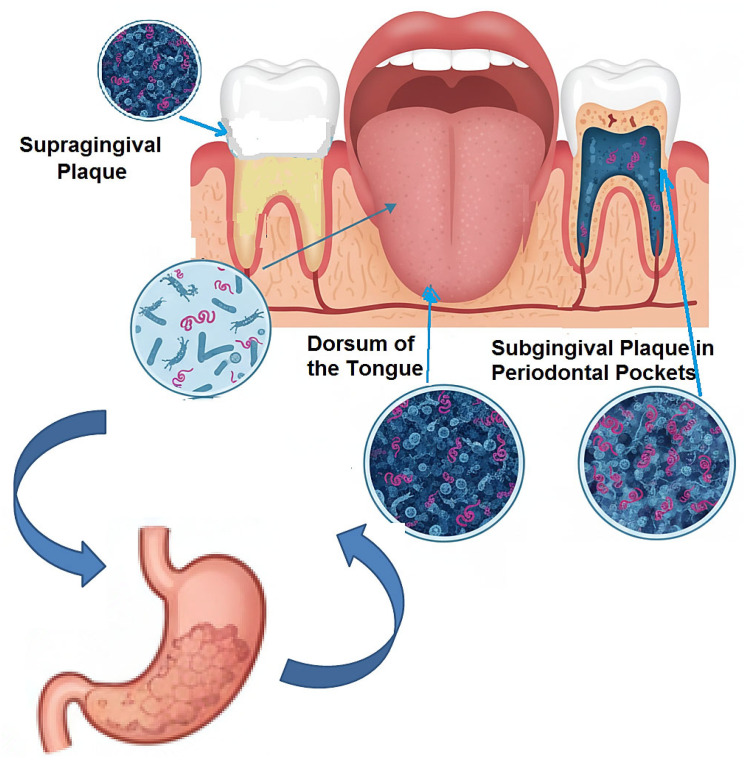
The Oral Cavity as an Extra-Gastric Reservoir.

The most established and extensively researched hypothesis linking the oral cavity to gastritis revolves around the role of *Helicobacter pylori* (*H. pylori*) [51,52,53]. While the gastric mucosa is the primary ecological niche for this pathogen, the persistent challenges of eradication failure, recrudescence, and reinfection following apparently successful triple or quadruple therapy have long suggested the existence of sanctuary sites outside the stomach [54,55,56]. The oral cavity, particularly the complex microbial biofilms of dental plaque and periodontal pockets, has emerged as the most plausible candidate for this extra-gastric reservoir [57,58,59], (Figure 2).

The initial evidence for this paradigm was built on the detection of *H. pylori* within the oral environment. Early studies utilizing culture techniques frequently isolated *H. pylori* from dental plaque and saliva, though with variable success rates, likely due to the fastidious nature of the bacterium and its potential transition to a viable-but-non-culturable (VBNC) state outside the stomach [60,61]. The advent of polymerase chain reaction (PCR) technology, targeting specific *H. pylori* genes (e.g., *ureA*, *glmM*, *16S rRNA*, *vacA*), provided more sensitive and conclusive evidence. A multitude of PCR-based studies have consistently detected *H. pylori* DNA in supragingival and subgingival plaque, saliva, and even the dorsum of the tongue, with prevalence rates ranging from 20% to over 80% in individuals with gastric infection [62,63,64,65]. This wide variation is attributable to differences in sampling techniques, DNA extraction methods, primer specificity, and the population studied.

Critically, the presence of *H. pylori* in the mouth is not merely incidental; it is significantly associated with oral health status. A compelling body of evidence indicates that the detection rate of oral *H. pylori* is markedly higher in individuals with periodontitis compared to those with healthy periodontium [30,66,67]. The subgingival plaque within periodontal pockets provides an ideal micro-environment: it is relatively protected from salivary flow and mechanical disruption, exhibits a reduced oxygen tension that may favor microaerophilic bacteria, and possesses a rich proteinaceous exudate (gingival crevicular fluid) that could serve as a nutrient source [68,69,70]. This association suggests that periodontal disease creates a conducive habitat for *H. pylori* colonization and persistence.

The clinical significance of the oral reservoir is profoundly illustrated in the context of eradication therapy. The standard triple therapy, comprising a proton pump inhibitor and two antibiotics, is highly effective against gastric *H. pylori* but achieves poor bioavailability in saliva and may not effectively penetrate mature dental biofilm [71,72,73,74]. This creates a therapeutic dilemma: while gastric colonization is cleared, the oral reservoir remains untouched. Several studies have demonstrated that the presence of *H. pylori* in dental plaque prior to treatment is a significant predictor of eradication failure [51,68,75]. The proposed mechanism is that following the cessation of antibiotics, the orally resident bacteria can be swallowed, successfully recolonizing the now-vacant gastric niche and leading to recrudescence of the infection. This hypothesis is supported by studies showing identical *H. pylori* strains, as determined by random amplified polymorphic DNA (RAPD) or whole-genome sequencing, in the plaque and stomach of the same patient pre- and post-treatment failure [17,36,76,77,78].

Further strengthening the reservoir hypothesis is interventional evidence. While still an emerging area of research, some studies have investigated the effect of adjunctive oral hygiene interventions on *H. pylori* eradication rates. For instance, a systematic review from 2025 that included clinical studies demonstrated that patients who received non-surgical periodontal treatment (NSPT), such as scaling and root planing, in conjunction with standard antibiotic therapy for *H. pylori* had a higher gastric eradication rate and a significantly lower recurrence rate than those who received antibiotic therapy alone [79]. This suggests that reducing the bacterial load in the oral cavity, a potential reservoir for *H. pylori*, can directly improve the long-term success of gastric treatment, providing a causal link between oral health and therapeutic outcomes.

However, the oral *H. pylori* paradigm is not without its controversies and complexities. A primary critique is the difficulty in culturing the bacterium from oral samples, leading some to argue that detected DNA may originate from dead cells or non-viable fragments shed from the stomach [80,81,82]. Furthermore, some highly sensitive studies have failed to find a significant oral presence, suggesting that the reservoir’s importance may vary between populations and individuals [83]. There is also ongoing debate about whether *H. pylori* is a true colonizer of the mouth or a transient passenger continually re-seeded from the stomach. Nevertheless, the cumulative weight of epidemiological, molecular, and clinical intervention evidence presents a persuasive case for the oral cavity, particularly in the context of periodontal disease, acting as a clinically relevant reservoir that compromises the long-term management of *H. pylori*-associated gastritis.

This classic paradigm provides an essential foundation, but it also serves as a springboard into a more complex and holistic understanding of the oral-gastric axis, which extends far beyond this single pathogen.

## 4. Beyond *H. pylori:* The Oral–Gut Axis in the Microbiome Era

**Figure 3:**
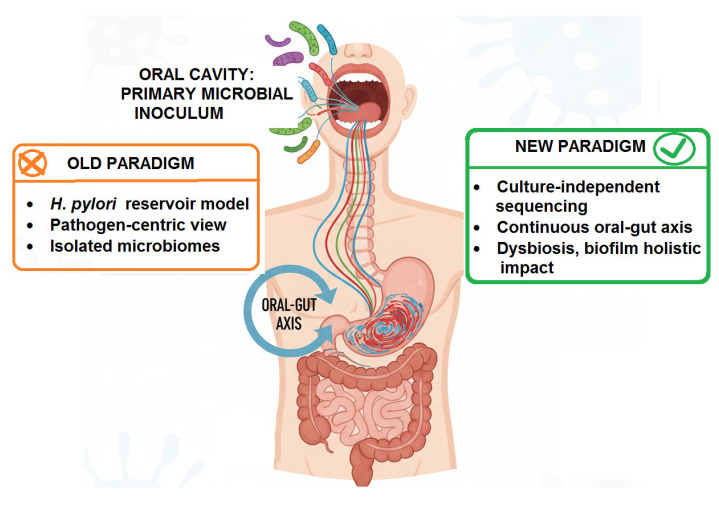
A Conceptual View of the Paradigm Shift in Oral–Gut Axis.

While the *H. pylori* reservoir model provides a strongly supported direct link, it represents a pathogen-centric view of a far more complex ecological dialog. The advent of culture-independent, high-throughput sequencing technologies has dismantled the notion of isolated microbiomes, revealing the existence of a continuous oral–gut axis wherein the oral cavity acts as a primary microbial inoculum for the entire gastrointestinal tract [84]. This paradigm shift moves the focus from a single pathogen to the collective impact of the entire oral microbial community, its state of dysbiosis, and its profound potential to influence gastric homeostasis and inflammation (Figure 3).

### 4.1. Oral Dysbiosis: More than Just Pathogen Overgrowth

Periodontitis and dental caries are no longer viewed simply as infections by specific bacteria but as canonical examples of dysbiosis, a pathological disruption of the symbiotic microbiome-host relationship [24,40,85]. In periodontal health, a diverse community of commensal bacteria is maintained in balance by host immunity [86]. The shift to dysbiosis is driven by ecological pressures (e.g., diet, inflammation) that select for a less diverse, more virulent microbiota [87,88,89,90]. Key periodontopathogens like *Porphyromonas gingivalis*, *Tannerella forsythia*, and *Treponema denticola* (the “red complex”) are not mere passengers but “keystone pathogens” that manipulate the host immune response (e.g., via subversion of complement and toll-like receptor signaling) to create a state of destructive inflammation that benefits themselves and other inflammophilic pathobionts [91,92].

This dysbiotic community is characterized by:

- Loss of beneficial taxa and overall diversity.
- Overgrowth of pro-inflammatory and proteolytic bacteria.
- Increased production of virulence factors (e.g., lipopolysaccharide (LPS), gingipains, fimbriae).
- A breached epithelial barrier in the periodontal pocket, facilitating the systemic dissemination of both bacteria and inflammatory mediators.

It is this entire dysbiotic consortium, not just *H. pylori*, that is swallowed continuously into the stomach [93,94].

### 4.2. Translocation and Survival: The Journey to the Stomach

The gastric acid barrier is a formidable first line of defense, but evidence suggests that oral microbes are far from passive victims of this harsh environment. Many oral bacteria possess inherent acid tolerance mechanisms. *Fusobacterium nucleatum*, a bridging organism in periodontal dysbiosis, can survive at a pH as low as 3.0 for several hours [95,96]. More importantly, bacteria within biofilms or embedded in food debris are afforded significant protection from acid shock [42,97]. The constant influx—approximately 1.5 L of saliva containing over 10^8^ bacteria per milliliter—ensures a substantial daily microbial inoculum that can overcome gastric clearance through sheer numbers [27,98].

Metagenomic sequencing studies have confirmed that the gastric fluid and mucosa of individuals, even in the absence of *H. pylori*, contain a detectable microbial signature of oral taxa, including *Streptococcus*, *Veillonella*, *Prevotella*, and *Granulicatella* [38,99,100,101]. This “oralization” of the gastric microbiome is significantly more pronounced in individuals with poor oral health, suggesting that a diseased oral cavity directly seeds the stomach with its dysbiotic microbiota [37,102,103,104].

### 4.3. Ecological Impact: Disrupting the Gastric Niche

**Figure 4:**
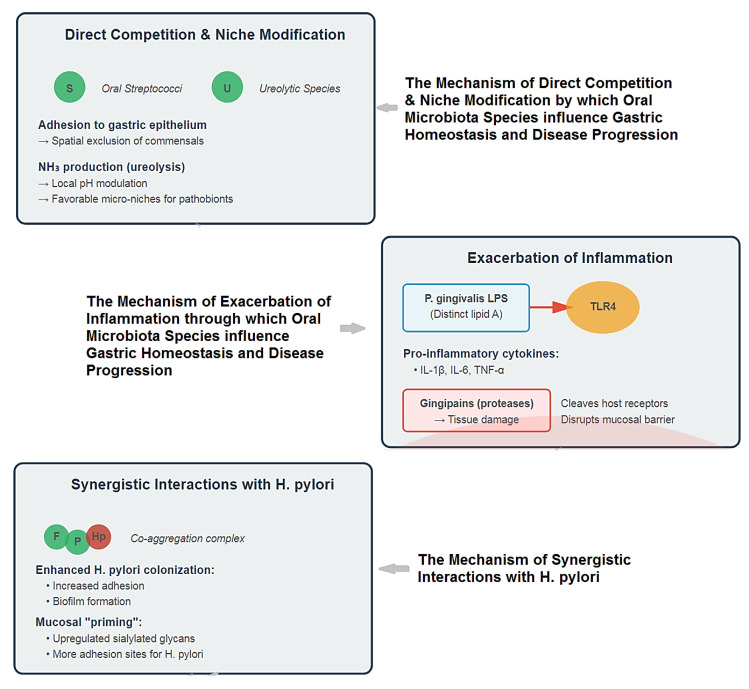
Multifaceted Mechanisms of Oral Microbiota in Gastric Pathogenesis.

The ecological impact of this constant seeding of oral bacteria on the gastric environment is an area of intense and novel research. The image below delineates the pathogenic interplay between oral microbiota and gastric mucosa, encompassing direct competition with niche modification, exacerbated inflammation, and synergistic interactions with *H. pylori* that collectively disrupt homeostasis and drive disease (Figure 4).

Thus, the proposed mechanisms extend far beyond simple colonization:

1. Direct Competition and Niche Modification: The influx of oral bacteria can compete with resident gastric microbes for space and nutrients. Some oral streptococci are adept at binding to gastric epithelial cells, potentially excluding beneficial commensals [105,106,107]. Furthermore, the metabolic activity of oral bacteria (e.g., production of ammonia by ureolytic species) could locally modulate pH, creating micro-niches that favor the growth of other acid-sensitive pathobionts [108] (Figure 3).
2. Exacerbation of Inflammation: This is perhaps the most significant mechanism. The gastric mucosa is in a constant state of low-grade exposure to swallowed oral microbes. In health, this may contribute to immune homeostasis. However, the swallowing of a *dysbiotic*, inflammation-primed oral microbiome delivers a heightened load of potent immunostimulatory molecules [109,110].
  - LPS from oral Gram-negative bacteria (e.g., *P. gingivalis*), which has distinct lipid A structures compared to enteric LPS, can activate Toll-like receptor 4 (TLR4) on gastric epithelial and immune cells, triggering the production of pro-inflammatory cytokines (IL-1β, IL-6, TNF-α) [111,112].
  - Bacterial virulence factors like gingipains from *P. gingivalis* are potent proteases that can directly damage tissue and cleave host cell surface receptors, further dysregulating immune responses and potentially disrupting gastric mucosal integrity [113,114,115], (Figure 3).
3. Synergistic Interactions with *H. pylori*: The oral-gastric axis may critically modulate *H. pylori* pathogenicity. Co-culture studies show that oral bacteria like *F. nucleatum* and *P. gingivalis* can enhance the adhesion and biofilm formation of *H. pylori* [108,116,117]. The inflammatory environment created by oral pathobionts could “prime” the gastric mucosa, upregulating adhesion receptors (e.g., sialylated glycans) that *H. pylori* exploits for colonization and amplifying the subsequent destructive host immune response to the gastric pathogen [43,118], (Figure 3).

This creates a vicious cycle where oral dysbiosis worsens gastric inflammation, which in turn may feedback to alter the oral environment. The table below outlines the key mechanisms by which the seeding of oral bacteria into the stomach can impact the gastric environment, moving beyond simple colonization to complex ecological and immunological interactions (Table 1).

This table positions the “oral-gastric axis” not as a passive conduit, but as an active driver of gastric ecology and disease. The state of the oral microbiome—symbiotic vs. dysbiotic—directly influences gastric health through these interconnected mechanisms, establishing oral health as a critical, modifiable factor in gastrointestinal disease prevention and management.

In conclusion, the microbiome era compels us to view the link between dental pathologies and gastritis not as a simple highway for a single pathogen, but as a complex, bustling waterway constantly transporting a microbial and inflammatory cargo. The state of that cargo, whether it is a balanced symbiotic community or a dysbiotic, inflammatory one, fundamentally shapes the ecology and immune landscape of the stomach. Critically, this constant seeding of a dysbiotic oral microbiome not only directly impacts the gastric niche but also serves as the primary instigator of the systemic inflammatory cascade, which we will explore in the following section. This broader perspective positions oral health not merely as a dental concern, but as a modifiable upstream factor in gastrointestinal health, with profound implications for preventive and therapeutic strategies.

## 5. The Inflammatory Bridge: Systemic Inflammation as a Mechanistic Link

Beyond the direct physical translocation of microbes, a second, potent pathway connects dental pathologies to gastritis: the dissemination of inflammatory mediators from the periodontium into the systemic circulation [119,120]. Periodontitis is not a localized infection but a chronic inflammatory disease with measurable systemic repercussions [48]. This low-grade, persistent inflammatory state can “prime” the host’s immune system, thereby lowering the threshold for inflammatory responses in distant organs, including the gastric mucosa [121]. This systemic inflammatory bridge provides a powerful mechanism by which oral dysbiosis can exacerbate gastric inflammation, even in the absence of direct bacterial colonization [122].

### 5.1. The Periodontium as a Factory of Inflammatory Mediators

The dysbiotic biofilm in periodontitis triggers a sustained and dysregulated host immune response. Resident cells (gingival epithelial cells, fibroblasts) and recruited immune cells (primarily neutrophils, macrophages, and lymphocytes) release a storm of pro-inflammatory cytokines, chemokines, and acute-phase proteins in an attempt to control the bacterial challenge [123,124].

Key players include:

- Interleukin-1β (IL-1β) and Tumor Necrosis Factor-α (TNF-α): Potent pro-inflammatory cytokines that drive bone resorption, activate endothelial cells, and stimulate the production of other cytokines [125,126].
- Interleukin-6 (IL-6): A pleiotropic cytokine that induces hepatic production of C-reactive protein (CRP) and promotes Th17 differentiation [127,128].
- Prostaglandin E2 (PGE2): A key mediator of pain, vasodilation, and bone destruction [129].

In healthy periodontium, this response is controlled and localized. In periodontitis, the response becomes chronic and excessive, leading to the collateral damage of periodontal tissues. Importantly, these inflammatory mediators enter the systemic circulation via the sulcular epithelium—an ulcerated, permeable tissue that is continuous with the gingival crevice and has a rich capillary network [130].

### 5.2. Measurable Systemic Inflammatory Burden

The systemic spillover from periodontitis is well-documented. Individuals with periodontitis consistently exhibit higher serum levels of CRP, IL-6, and other inflammatory markers compared to periodontal healthy controls [45,46,131,132]. This is not merely an association; successful periodontal treatment (e.g., scaling and root planning) has been shown to significantly reduce these systemic levels of inflammation, demonstrating a direct causal link [133,134].

This chronic, low-grade systemic inflammation creates a heightened state of immune alertness throughout the body. Circulating monocytes and neutrophils from periodontitis patients display a “primed” phenotype, characterized by enhanced production of reactive oxygen species (ROS) and cytokines upon secondary stimulation [135,136].

### 5.3. Exacerbation of Gastric Inflammation: Mechanisms of Action

This systemically disseminated inflammatory burden can exacerbate gastritis through several interconnected mechanisms, which prime the host immune response, amplify recruitment of inflammatory cells, and compromise gastric mucosal defense (summarized in Table 2).

The following paragraphs detail the evidence for these pathways.

Priming of Gastric Mucosal Immune Cells: The gastric mucosa is populated by resident immune cells, including macrophages and dendritic cells. Constant exposure to elevated levels of systemic IL-1β, TNF-α, and IL-6 can prime these cells, altering their response threshold. When these pre-activated cells encounter a gastric trigger—such as *H. pylori*, Non-Steroidal Anti-Inflammatory Drugs (NSAIDs), or even swallowed oral pathobionts—they mount an exaggerated inflammatory response. This leads to increased local production of inflammatory cytokines, greater tissue damage, and more severe gastritis [137,138,139].

Endothelial Activation and Leukocyte Recruitment: Systemic inflammatory cytokines, particularly TNF-α and IL-1β, activate the vascular endothelium throughout the body. In the gastric microvasculature, this activation upregulates adhesion molecules (e.g., E-selectin, ICAM-1, VCAM-1), facilitating the enhanced recruitment of circulating primed neutrophils and monocytes into the gastric tissue [140,141]. This amplified influx of inflammatory cells directly contributes to tissue damage through the release of proteolytic enzymes (e.g., matrix metalloproteinases) and ROS [142].

Synergy with *H. pylori* Pathogenesis: The systemic inflammatory milieu can directly interact with *H. pylori* infection. *H. pylori* itself induces a pro-inflammatory response in the stomach, primarily through the cag Pathogenicity Island (cagPAI) which activates NF-κB signaling. Systemic inflammation from periodontitis can synergize with this pathway. For instance, TNF-α is a powerful activator of NF-κB. The combined effect of *H. pylori* virulence factors and systemically elevated TNF-α could lead to a hyper-activation of NF-κB, resulting in a massive overproduction of gastric IL-8 and a significantly more robust neutrophilic infiltrate, thereby amplifying the severity of *H. pylori*-induced gastritis [137,143,144].

Impairment of Mucosal Defense and Repair: Chronic systemic inflammation can compromise the intrinsic defense mechanisms of the gastric mucosa. TNF-α, for example, can inhibit the proliferation of gastric epithelial cells and impair mucosal healing, leaving the tissue more vulnerable to injury and delaying repair processes [145,146]. This condition renders the gastric mucosa more susceptible to injury.

In summary, the inflammatory bridge model posits that the oral cavity acts as an endocrine-like source of inflammation. The chronic inflammatory lesion in the periodontium systemically elevates key mediators that alter the host’s immune set-point. This, in turn, predisposes the gastric mucosa to more severe inflammatory outcomes upon encountering any challenge, be it infectious, chemical, or dietary [147,148,149]. This mechanism operates in parallel to and can synergize with the direct microbial translocation pathways, and may even be exacerbated by the concurrent loss of protective, microbiome-derived signaling molecules such as nitric oxide. This mechanism operates in parallel to and can synergize with the direct microbial translocation pathways, providing a comprehensive explanation for the observed epidemiological links between periodontitis and gastritis. It underscores that treating oral inflammation may be a viable strategy for modulating systemic inflammatory burden and mitigating its impact on gastrointestinal health.

## 6. Other Potential Mechanisms: Nitrate Metabolism, Molecular Mimicry, and Salivary Factors

While microbial translocation and systemic inflammation represent the primary mechanistic pathways, several other intriguing mechanisms may contribute to the link between oral health and gastritis. These ancillary pathways highlight the profound complexity of the oral-gastric axis and offer additional avenues for research.

### 6.1. Nitrate Metabolism and Gastric Mucosal Defense

**Figure 5:**
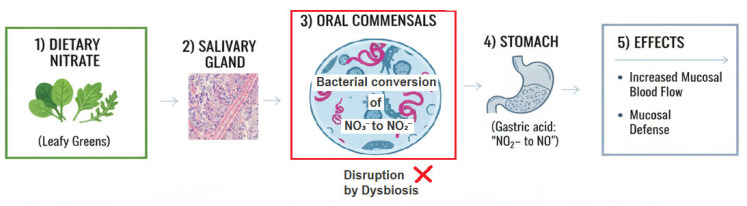
Dietary Nitrate Metabolism Pathway.

The nitrate-nitrite-nitric oxide (NO) pathway is a fundamental signaling and defense system, and the oral microbiome plays an indispensable role as its primary activator. Dietary nitrate (from leafy green vegetables) is absorbed and concentrated in saliva. Oral commensal bacteria, particularly on the dorsum of the tongue, possess nitrate reductase enzymes that reduce nitrate (NO_3_^−^) to nitrite (NO_2_^−^) [150,151]. Upon swallowing, this nitrite encounters the acidic gastric environment, where it is non-enzymatically reduced to potent vasodilator nitric oxide (NO) [152], (Figure 5).

The figure above is illustrating the enterosalivary nitrate-nitrite-NO pathway:

1. Dietary Nitrate—Intake from leafy greens and beetroot
2. Salivary Gland—Active concentration of nitrate (10–20× plasma levels) via the sialin transporter
3. Oral Commensals—Bacterial conversion of NO_3_^−^ to NO_2_^−^ by nitrate-reducing bacteria in tongue biofilm, with an “X” mark indicating disruption by dysbiosis
4. Stomach—Gastric acid-mediated conversion of NO_2_^−^ to NO and other nitrogen oxides
5. Physiological Effects—Increased mucosal blood flow and enhanced mucosal defense mechanisms

The red “X” over the oral step emphasizes how antiseptic mouthwash or oral dysbiosis can disrupt this critical bacterial conversion step, thereby reducing downstream NO production and its beneficial cardiovascular and gastrointestinal effects. This pathway demonstrates the important symbiotic relationship between dietary nitrate, oral microbiota, and systemic health.

This pathway is critically important for gastric health:

- Mucosal Blood Flow: NO induces vasodilation, increasing blood flow to the gastric mucosa, which is essential for maintaining the mucosal barrier, delivering oxygen and nutrients, and supporting repair mechanisms [153,154].
- Mucosal Defense: NO exhibits antimicrobial properties against a range of pathogens and can modulate immune responses [155].

The Periodontitis Disruption: Periodontal dysbiosis can drastically alter this beneficial process. The shift in the oral microbiome from a health-associated, nitrate-reducing community to a disease-associated, proteolytic one may impair the oral reduction of nitrate to nitrite. This reduction in bioavailable nitrite could lead to decreased gastric NO production, compromising mucosal blood flow and defense, and thereby increasing susceptibility to injury and inflammation, including *H. pylori*-associated damage [156,157]. Thus, poor oral health may not just add harmful elements, but also subtract an essential protective one.

### 6.2. Molecular Mimicry and Autoimmunity

Molecular mimicry occurs when microbial antigens share structural similarities with host self-antigens, potentially leading to the production of cross-reactive antibodies that attack host tissues. This mechanism is well-established in rheumatic heart disease following streptococcal infection [158].

There is preliminary evidence to suggest a similar process could play a role in certain forms of gastritis, particularly autoimmune gastritis (AIG). AIG is characterized by autoantibodies against parietal cell components, including the H^+^/K^+^ ATPase proton pump. Some oral bacteria possess antigens that may mimic these host structures. For instance, antibodies generated against *H. pylori* or other oral pathogens might cross-react with gastric epithelial cells, initiating or perpetuating an autoimmune inflammatory response [159,160,161].

While this link is more speculative and requires further validation, it represents a fascinating potential mechanism by which oral infections could trigger specific autoimmune responses against the stomach.

### 6.3. The Role of Saliva and Its Constituents

Saliva is far more than a simple transport medium; it is a complex biologic fluid whose composition is altered by oral disease, and these changes can directly influence the gastric environment [162,163]:

- Altered Buffering Capacity: Saliva is a primary buffer for gastric acid refluxed into the esophagus and oral cavity. Hyposalivation or altered composition in individuals with poor oral health may impair this neutralizing capacity, potentially prolonging acid contact time with esophageal and oropharyngeal tissues [27]. While this more directly impacts Gastroesophageal Reflux Disease (GERD), esophageal inflammation can have downstream effects on the gastric cardia.
- Immunological Components: Saliva contains numerous antimicrobial and immunomodulatory factors, including immunoglobulins (e.g., secretory IgA), lactoferrin, lysozyme, and histatins. The quality and quantity of these components can be affected by oral inflammation. For example, levels of certain protective proteins may decrease, while inflammatory mediators like cytokines (IL-1β, IL-6) from the gingival crevicular fluid increase in the saliva of individuals with periodontitis [29,164,165]. The swallowing of this “inflammatory saliva” provides a direct route for these mediators to contact the gastric mucosa.
- Matrix Metalloproteinases (MMPs): Periodontitis is associated with dramatically elevated levels of active MMPs (e.g., MMP-8, MMP-9) in saliva, released by neutrophils and fibroblasts to degrade collagen in periodontal tissues [166,167,168]. Upon swallowing, these proteolytic enzymes could theoretically contribute to the degradation of the gastric epithelial basement membrane and extracellular matrix, weakening mucosal integrity and promoting inflammation.

**Figure 6:**
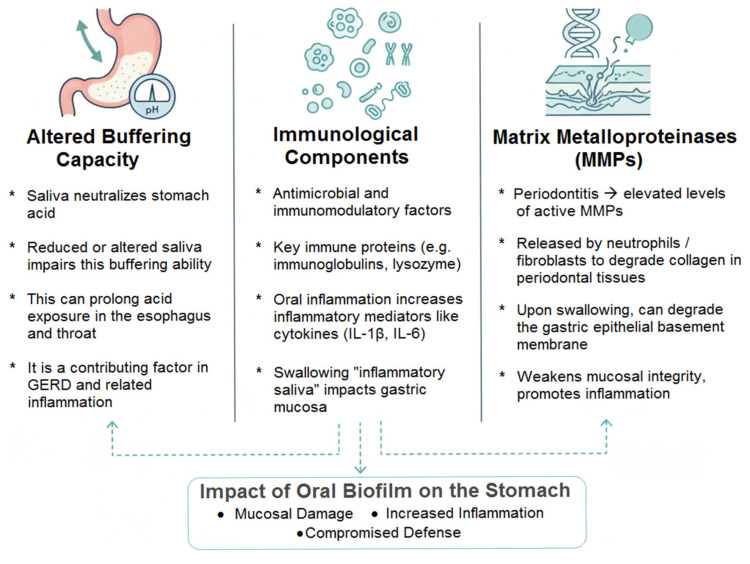
The Role of Saliva and its Constituents.

The figure below aims to represent the impact of saliva and its constituents on the gastric environment (Figure 6).

In conclusion, these ancillary mechanisms—the impairment of a protective nitrate cycle, the potential triggering of autoimmunity, and the alteration of saliva’s protective properties—collectively paint a picture of an oral-gastric relationship that is multifaceted. They suggest that the impact of oral biofilm on the stomach is not monolithic but may occur through a symphony of direct, indirect, metabolic, and immunological effects, further strengthening the argument for integrative management of oral and gastrointestinal health.

## 7. Discussion: The Oral-Gastric Axis: A Causal Link or a Plausible Illusion? A Critical Debate

The mechanistic pathways linking the oral and gastric environments are compelling, yet the ultimate clinical significance hinges on robust human evidence. This section moves beyond a simple synthesis to engage in the central, unresolved debate: Is the oral-gastric axis a modifiable causal pathway or merely an epiphenomenon of shared risk factors? It thoroughly evaluates the evidence for both positions, aiming to provide a balanced perspective on this evolving paradigm.

### 7.1. The Case for Causality

Proponents of a causal relationship point to converging lines of evidence from epidemiology, microbiology, and intervention that form a coherent, multi-faceted narrative. It is important to note that these mechanistic pathways—direct translocation, systemic inflammation, and ancillary metabolic functions—are not isolated but are deeply interconnected and often act in concert to drive gastric pathology.

#### 7.1.1. Consistent Epidemiological Association

A growing body of evidence from large-scale, prospective cohorts underscores a link between poor oral health and an elevated risk of gastrointestinal cancers, though the specific associations vary by cancer site and population. A nationwide cohort study in Sweden by Ruan et al. provides some of the strongest evidence to date, demonstrating that periodontitis was associated with an 11% increased risk of gastric cancer and a 25% increased risk of cardia gastric cancer specifically [169]. This study further identified a clear dose–response relationship, where having fewer remaining teeth was progressively associated with a higher risk of both gastric cancer subtypes. The findings were bolstered by sibling-controlled analyses, which reinforced the association while accounting for shared genetic and environmental familial factors. However, the relationship appears to be cancer-specific, as illustrated by a prospective analysis of the UK Biobank by Jordão et al. [170]. That study found no overall association between self-reported poor oral health (painful gums, bleeding gums, loose teeth) and the risk of most gastrointestinal cancers, including esophageal, gastric, and colorectal cancers. Notably, it did identify a significant, site-specific increase in risk for hepatobiliary cancers, with the association being strongest for hepatocellular carcinoma (HR = 1.75). Complementing these findings, a recent case–control study from Southwest China by Luo et al. confirmed significant associations between periodontitis and upper gastrointestinal cancers, identifying it as an independent risk factor for esophageal cancer (OR = 2.810), colon cancer (OR = 2.330), and rectal cancer (OR = 2.730) even after extensive adjustment for confounders [171]. Importantly, Luo et al. also demonstrated that periodontitis was significantly associated with distant metastasis in rectal cancer, with severe periodontitis conferring a markedly higher risk (aHR = 10.138), suggesting a role for oral health in cancer progression.

#### 7.1.2. The Oral *H. pylori* Reservoir Hypothesis: A Reinforced Causal Argument

A key pillar of the causal argument is the oral cavity acting as a significant reservoir for *H. pylori*, which has profound implications for gastric infection and reinfection. The most compelling evidence comes from a recent systematic review and meta-analysis by Anand et al. (2025), which synthesized data from 27 observational studies and 2408 participants [59]. Their work conclusively demonstrated that the presence of gastric *H. pylori* infection was significantly higher among patients with *H. pylori* in their dental plaque, with a pooled odds ratio of 3.80 (95% CI: 2.24–6.43). This robust statistical association strongly suggests that dental plaque can serve as an extra-gastric reservoir from which the microorganism can recolonize the gastric mucosa after eradication therapy. A critical advance in validating this reservoir hypothesis was the successful culturing of viable *H. pylori* from the oral cavity. As reviewed by Zhang et al. (2022), since the initial isolation by Krajden et al. in 1989, a limited number of studies have successfully cultured *H. pylori* from dental plaque, saliva, and even dental pulp samples [33,172]. The ability to culture the bacterium confirms its metabolic activity and viability in the oral environment, moving beyond mere DNA detection. Furthermore, research into the broader oral ecosystem suggests that specific biofilms and inter-microbial interactions may facilitate *H. pylori* colonization and persistence. Zhang et al. (2022) elaborate that the oral cavity is not an ideal habitat for *H. pylori* on its own; however, the bacterium can survive by integrating into dental plaque biofilms [33]. Within these structures, bacteria like *Fusobacterium nucleatum* can act as a bridge for co-aggregation, and streptococci may consume oxygen to create a more favorable microaerophilic environment. Moreover, a remarkable survival strategy involves *H. pylori* invading yeast cells such as *Candida albicans*, which provides an intracellular niche that protects it from environmental stresses and antibiotics. This complex interplay with the oral microbiome helps explain the persistence of *H. pylori* in the mouth and its role in recalcitrant gastric infections.

#### 7.1.3. Direct Translocation of Oral Pathobionts

Beyond *H. pylori*, the broader oral and gastric microbiome is implicated in gastric pathogenesis. Metagenomic sequencing studies consistently show that oral microbes, including members of the genera *Peptostreptococcus*, *Streptococcus*, and particularly *Fusobacterium nucleatum*, can colonize the gastric mucosa, with their abundance often increased in gastric cancer tissues [173,174]. *F. nucleatum* has emerged as a prime candidate for a pro-tumorigenic role. Work by Coker et al. demonstrated its association with microbial dysbiosis in gastric carcinogenesis, while Sorino et al. provided direct mechanistic evidence showing that *F. nucleatum* promotes gastric cancer progression by modulating the tumor microenvironment, specifically by activating immune checkpoint pathways to suppress anti-tumor immunity [175]. This mechanism mirrors its extensively documented role in other gastrointestinal cancers [176,177].

#### 7.1.4. The Systemic Inflammation Pathway

A compelling, though still evolving, body of evidence suggests that periodontitis is not merely a localized oral issue but a significant contributor to systemic disease, particularly cancer. The chronic, low-grade inflammation it seeds throughout the body creates a fertile ground for tumorigenesis at distant sites. This is not a settled consensus but a hypothesis strengthened by key findings. Researchers like Slade et al. and D’Aiuto et al. have provided foundational evidence, demonstrating that periodontal disease directly elevates systemic inflammatory markers like CRP and IL-6, and that treating the oral infection mitigates this response [45,178]. The critical link between this inflammatory milieu and cancer is powerfully articulated by Grivennikov et al., who detail how cytokines precisely like those elevated in periodontitis can drive protumorigenic processes [179]. The most provocative evidence, however, comes from work like that of Salazar et al. and reviews by Zhou et al., which move beyond correlation to implicate specific periodontal pathogens, such as *Porphyromonas gingivalis*, in the pathogenesis of gastric precancerous lesions themselves [180,181]. While questions of absolute causality remain, the collective weight of this research positions periodontal health as a plausible and modifiable risk factor in the broader landscape of cancer prevention.

#### 7.1.5. Preliminary Interventional Support

The proposition that periodontal therapy can directly influence gastrointestinal health finds its most compelling, yet nuanced, support in interventional studies. Proponents of this oral-systemic link often cite the robust prospective randomized trial by Tongtawee et al., which demonstrated that while adjunctive periodontal therapy did not significantly boost the initial *H. pylori* eradication rate, it profoundly and significantly reduced the recurrence of gastric infection [182]. This is an important distinction, as it strongly implicates the oral cavity as a reservoir for reinfection, a theory bolstered by their finding of a close relationship between *H. pylori* in saliva and in the stomach. Synthesizing the available evidence, the systematic review by Inchingolo et al. concludes that there is indeed evidence that patients with *H. pylori* infection benefit from non-surgical periodontal treatment (NSPT) and even suggests it might be included in future eradication guidelines [79]. However, this is where the debate sharpens. A skeptical view must highlight the caveats that Inchingolo et al. [79] themselves underscore: the current body of evidence is limited by small sample sizes, short follow-up periods, and a scarcity of large-scale RCTs. Therefore, while the findings from Tongtawee et al. [182] on preventing recurrence are powerful and mechanistically clear, the scientific community largely agrees with the systematic review’s conclusion that more evidence is required before this becomes a standard clinical mandate. The relationship is modifiable in theory, but its universal application is not yet fully supported by the evidence.

### 7.2. The Case for Skepticism

Despite the compelling narrative, skeptics rightly argue that the evidence for causality is overstated and vitiated by methodological limitations that are difficult to overcome.

#### 7.2.1. The Intractable Problem of Confounding

The most potent counterargument against a causal link between periodontitis and gastric cancer is the profound and likely insurmountable challenge of confounding. The established risk factors for periodontitis—low socioeconomic status, smoking, poor diet, and alcohol consumption—are, in many cases, the identical risk factors for gastric cancer. This creates a perfect storm for spurious conclusions in observational studies. A critical appraisal of the literature confirms that residual confounding is a near certainty. This pervasive confounding is likely responsible for the inconsistent findings across large studies. For instance, the prospective analysis of the UK Biobank by Jordão et al. found no overall association between self-reported poor oral health and the risk of gastric cancer, highlighting how the relationship may not be robust when examined in different populations and with different exposure measurements [170]. As Meyer et al. (2017) highlight in their systematic review, while studies show an association, it remains difficult to exclude confounding and bias as explanations for the findings due to these shared risk factors [183]. This is not a minor issue; it is paramount. The work of Ahn et al. (2012) correctly underscores that oral and GI cancers share a ‘common soil’ of powerful environmental risk factors, particularly smoking [184]. Therefore, until data is presented that fully and rigorously accounts for this pervasive confounding, which even the most sophisticated multivariate models can only partially alleviate, any claimed direct, causal link between the oral microbiome and gastric carcinogenesis remains speculative, not proven.

#### 7.2.2. The Cross-Sectional Conundrum and Reverse Causality

The claim that oral bacteria directly cause gastric cancer is critically weakened by the Cross-Sectional Conundrum and the strong likelihood of Reverse Causality. Most human evidence comes from cross-sectional studies: single snapshots comparing cancer patients to healthy controls. This design is fundamentally unable to determine if oral bacteria are the cause or the consequence of the disease. The compelling alternative is reverse causality. Gastric carcinogenesis follows a known sequence: from healthy tissue to chronic gastritis, atrophy, and hypochlorhydria (low stomach acid). This dismantles the stomach’s primary microbial barrier. It is highly probable that this loss of acid allows for retrograde colonization by oral bacteria. This is not just speculation; it is supported by evidence:

- Hypochlorhydria Comes First: Ferreira et al. (2018) showed that patients with precancerous hypochlorhydria already have a stomach microbiome enriched with oral taxa, proving this shift occurs before cancer [174].
- Animal Model Proof: Lofgren et al. (2011) demonstrated that the loss of acid-secreting cells directly enables gastric colonization by commensal bacteria, establishing a clear cause-and-effect [185].
- Correlation with Stage: Coker et al. (2018) and others find that oral bacteria increase step-wise with disease severity, peaking in advanced precancerous states, marking them as passengers in a permissive environment [173].

Therefore, the evidence aligns perfectly with the interpretation that altered stomach physiology is the primary event, and the influx of oral bacteria is a secondary effect. Therefore, the temporal sequence essential for establishing causality remains unproven, and the observed enrichment of oral bacteria in gastric tumors may be a consequence of the diseased gastric environment rather than its cause.

#### 7.2.3. Microbiome Complexity and the “Passenger” Hypothesis

The complexity of the microbiome is a challenge, but modern sequencing is clarifying the picture. While the overall abundance of oral taxa in a healthy stomach is low, specific pathogens like *Fusobacterium nucleatum* are consistently enriched within gastric tumor tissue, suggesting a non-random, tropic relationship. A growing body of evidence from mechanistic studies demonstrates that these bacteria are not passive ‘passengers’ but active ‘drivers’ of disease, capable of promoting tumorigenesis and chemoresistance by modulating host signaling pathways [174,186,187]. The difficulty in culturing these bacteria from early-stage lesions does not negate their role; it may reflect technical limitations or support a ‘hit-and-run’ model of pathogenesis where an initial trigger sets off a lasting oncogenic cascade.

#### 7.2.4. Inconsistent Interventional Evidence

The interventional evidence is critically weak and marked by directly conflicting outcomes. The trial by Tongtawee et al. found that periodontal therapy significantly reduced *H. pylori* recurrence but did not improve the initial eradication rate [182]. This contrasts with the conclusion of the systematic review by Inchingolo et al., which suggests a benefit for NSPT but itself highlights the scarcity of large-scale RCTs and the low quality of the existing evidence [79]. This inconsistency suggests the relationship is not straightforward and may depend on intervention intensity, timing, and patient population. The claim that improving oral health directly improves gastric outcomes is not robustly supported by high-level evidence, which is dominated by small, underpowered pilot studies and conflicting results from randomized controlled trials. This inconsistency suggests the relationship is not straightforward and may depend on intervention intensity, timing, and patient population.

#### 7.2.5. Limitations in Measuring Exposure

The measurement of periodontal disease exposure is hampered by significant methodological heterogeneity and potential for misclassification. Key limitations include the use of variable case definitions, ranging from self-report to clinical measurement, and the reliance on tooth loss as a proxy. Furthermore, the application of the 2018 Classification of Periodontitis has been inconsistent, a problem exacerbated by variability in examiner training, experience, and the use of diagnostic aids [188]. Consequently, methodological reviews consistently emphasize the need for standardized training and implementation tools to improve diagnostic accuracy and reliability.

### 7.3. Synthesis and Future Research: Resolving the Debate

The current evidence paints a complex picture, insufficient to declare a definitive victory for either side. The sheer volume of consistent epidemiological signals, coupled with plausible mechanisms and positive interventional trends, is too compelling to dismiss outright. However, the skepticism grounded in confounding, microbiome complexity, and inconsistent trials is valid and necessary for scientific rigor. The path to resolving this debate lies in future research designed to address these criticisms directly.

1. Next-Generation Targeted RCTs: Large, multi-center, double-blind RCTs are needed, comparing intensive, subgingival periodontal therapy against a sham control. These trials must use long-term *H. pylori* recurrence and gastric histologic improvement as primary endpoints, moving beyond short-term eradication rates.
2. Deep Mechanistic Embedding: These trials must embed correlative studies using multi-omics approaches (e.g., metagenomics, metatranscriptomics, and metabolomics) to track the flux of oral bacteria to the stomach and their functional impact. This will help move beyond correlation to mechanism and identify which patients are most likely to benefit.
3. Sophisticated Observational Analyses: Employing methods like Mendelian randomization could help triangulate causality by using genetic instruments for periodontitis susceptibility to assess its effect on gastric cancer risk, thereby mitigating confounding.

Conclusion of the Debate: While the “plausible illusion” of confounding remains a serious concern, the weight of evidence is gradually shifting towards a causal, and more importantly, a modifiable relationship. The oral-gastric axis, particularly involving *H. pylori* and specific pathobionts like *F. nucleatum*, should be considered a highly plausible and promising frontier for intervention. The burden of proof now lies with researchers to rigorously demonstrate through mechanistic trials outlined above that improving oral health translates into a tangible, long-term benefit for gastric health.

### 7.4. Limitations of This Review

While this review has sought to provide a comprehensive and critical synthesis, it is subject to several inherent limitations. Firstly, as a narrative review, it carries a potential for selection bias, unlike a protocol-driven systematic review. Secondly, the conclusions are constrained by the limitations pervasive in the primary literature, including a preponderance of cross-sectional data, inconsistent definitions of exposure and outcome, and inadequate control for confounding. Thirdly, while the mechanistic pathways were significantly discussed, their direct clinical translation remains inferential, and the current interventional evidence is too limited to firmly bridge this gap. Finally, the rapid evolution of microbiome science means this review represents a snapshot in time, and new studies with advanced technologies will undoubtedly refine the current understanding.

### 7.5. Clinical Implications

Despite the need for more definitive evidence, the current body of knowledge carries significant implications for clinical practice, advocating for a paradigm shift towards integrated care.

- Interdisciplinary Collaboration and Screening: The management of patients with chronic gastritis, particularly cases of *H. pylori* recurrence, should involve collaboration between gastroenterologists and dental professionals. A simple oral health assessment, including inquiries about gum bleeding, tooth mobility, and the time since the last dental examination, should be considered in the gastroenterological history-taking for high-risk patients. This low-cost step can identify individuals who may benefit from a formal dental referral.
- Oral Health as a Modifiable Risk Factor in *H. pylori* Management: For patients with concurrent *H. pylori* infection and periodontitis, non-surgical periodontal therapy could be considered a potential adjunct to standard eradication therapy. Based on emerging evidence, a pragmatic approach could be to schedule NSPT either shortly before or concurrently with the initiation of *H. pylori* eradication therapy. This timing aims to reduce the oral bacterial load and potential reservoir effect, potentially improving long-term eradication success and reducing recurrence. Promoting good oral hygiene may be a simple, cost-effective strategy to reduce systemic inflammatory burden.
- Patient Education and Empowerment: Patients should be educated about the oral-systemic connection, empowering them to take an active role in their overall well-being and improving adherence to both dental and medical treatments.

## 8. Conclusions

The intricate dialog between the oral cavity and the stomach, once a speculative concept, is now supported by a compelling, if not yet definitive, convergence of evidence. This review has synthesized the multifaceted pathways linking dental pathologies to gastritis. While definitive proof of causality awaits more rigorous data, the existing evidence presents a compelling narrative. The consistent epidemiological signals and plausible biological mechanisms are counterbalanced by significant methodological challenges, including confounding and the possibility of reverse causality. Therefore, the state of the oral microbiome is best considered a plausible and modifiable contributor to gastric health, rather than a definitive causative agent.

## Figures and Tables

**Table 1:** Oral-gastric axis as an active driver of gastric ecology and disease [105,106,107,108,109,110,111,112,113,114,115,116,117,118].

Mechanism	Specific Examples/Molecules Involved	Consequence
Direct Competition and Niche Modification	Oral Streptococci: Adhesion to gastric epithelial cells. Ureolytic Species: Production of ammonia.	Competes with and excludes resident gastric commensals for space and nutrients. Locally modulates pH, creating micro-niches favorable for acid-sensitive pathobionts.
Exacerbation of Inflammation	LPS from *P. gingivalis*: Distinct lipid A structure. Gingipains: Potent proteases from *P. gingivalis*.	Activates TLR4 on gastric cells, triggering pro-inflammatory cytokines (IL-1β, IL-6, TNF-α). Directly damages tissue, cleaves host cell receptors, and dysregulates immune responses, disrupting mucosal integrity.
Synergistic Interactions with *H. pylori*	*F. nucleatum*, *P. gingivalis*: Co-aggregation with *H. pylori*. Inflammatory Environment: Upregulation of host receptors (e.g., sialylated glycans).	Enhances *H. pylori* adhesion and biofilm formation. “Primes” the gastric mucosa, providing more adhesion sites for *H. pylori* and amplifying the destructive host immune response. Creates a vicious cycle of inflammation.

**Table 2:** The Systemic Inflammatory Bridge: Mechanisms Linking Periodontitis to Exacerbated Gastritis.

Mechanism of Action	Key Mediators/Cells Involved	Consequence in the Gastric Mucosa
Priming of Gastric Mucosal Immune Cells	Systemically elevated IL-1β, TNF-α, IL-6 [45,46,131]	Pre-activated gastric macrophages and dendritic cells mount an exaggerated inflammatory response to triggers like *H. pylori*, increasing local cytokine production and tissue damage [137,138,139].
Endothelial Activation and Leukocyte Recruitment	TNF-α, IL-1β; Circulating “primed” neutrophils and monocytes [135,136,140]	Upregulation of endothelial adhesion molecules (e.g., ICAM-1) in gastric microvasculature enhances recruitment of inflammatory cells, which release proteases (MMPs) and ROS, causing collateral damage [141,142].
Synergy with *H. pylori* Pathogenesis	Systemic TNF-α; *H. pylori* cagPAI (NF-κB pathway) [126,143]	Synergistic hyper-activation of NF-κB leads to overproduction of chemokines like IL-8, resulting in a significantly amplified neutrophilic infiltrate and more severe gastritis [137,144].
Impairment of Mucosal Defense and Repair	TNF-α [145,146]	Direct inhibition of gastric epithelial cell proliferation and impairment of mucosal healing pathways, rendering the tissue more susceptible to injury and delaying repair.

## Abbreviations

The following abbreviations are used in this manuscript:

*H. pylori*	*Helicobacter pylori*
NSPT	non-surgical periodontal treatment
PCR	Polymerase Chain Reaction
NSAIDs	Non-Steroidal Anti-Inflammatory Drugs
NO	nitric oxide
GERD	Gastroesophageal Reflux Disease
AIG	autoimmune gastritis
